# Identification of super-infected *Aedes triseriatus *mosquitoes collected as eggs from the field and partial characterization of the infecting La Crosse viruses

**DOI:** 10.1186/1743-422X-7-76

**Published:** 2010-04-22

**Authors:** Sara M Reese, Eric C Mossel, Meaghan K Beaty, Eric T Beck, Dave Geske, Carol D Blair, Barry J Beaty, William C Black

**Affiliations:** 1 Arthropod-Borne and Infectious Diseases Laboratory, Department of Microbiology, Immunology and Pathology, Colorado State University, Fort Collins, Colorado 80523-1692, USA; 2 Current address: Colorado Department of Public Health and Environment, 4300 Cherry Creek Drive South, Denver, CO 80246, USA; 3 Current address: Midwest Respiratory Virus Program, Department of Pediatric Infectious Diseases, Medical College of Wisconsin, Milwaukee, WI 53226, USA; 4 La Crosse County Health Department, La Crosse, WI 54601-3228, USA

## Abstract

**Background:**

La Crosse virus (LACV) is a pathogenic arbovirus that is transovarially transmitted by *Aedes triseriatus *mosquitoes and overwinters in diapausing eggs. However, previous models predicted transovarial transmission (TOT) to be insufficient to maintain LACV in nature.

**Results:**

To investigate this issue, we reared mosquitoes from field-collected eggs and assayed adults individually for LACV antigen, viral RNA by RT-PCR, and infectious virus. The mosquitoes had three distinct infection phenotypes: 1) super infected (SI+) mosquitoes contained infectious virus, large accumulations of viral antigen and RNA and comprised 17 of 17,825 (0.09%) of assayed mosquitoes, 2) infected mosquitoes (I+) contained no detectable infectious virus, lesser amounts of viral antigen and RNA, and comprised 3.7% of mosquitoes, and 3) non-infected mosquitoes (I-) contained no detectable viral antigen, RNA, or infectious virus and comprised 96.21% of mosquitoes. SI+ mosquitoes were recovered in consecutive years at one field site, suggesting that lineages of TOT stably-infected and geographically isolated *Ae. triseriatus *exist in nature. Analyses of LACV genomes showed that SI+ isolates are not monophyletic nor phylogenetically distinct and that synonymous substitution rates exceed replacement rates in all genes and isolates. Analysis of singleton versus shared mutations (Fu and Li's F*) revealed that the SI+ LACV M segment, with a large and significant excess of intermediate-frequency alleles, evolves through disruptive selection that maintains SI+ alleles at higher frequencies than the average mutation rate. A QTN in the LACV NSm gene was detected in SI+ mosquitoes, but not in I+ mosquitoes. Four amino acid changes were detected in the LACV NSm gene from SI+ but not I+ mosquitoes from one site, and may condition vector super infection. **In contrast to NSm**, the NSs sequences of LACV from SI+ and I+ mosquitoes were identical.

**Conclusions:**

SI+ mosquitoes may represent stabilized infections of *Ae. triseriatus *mosquitoes, which could maintain LACV in nature. A gene-for-gene interaction involving the viral NSm gene and a vector innate immune response gene may condition stabilized infection.

## Background

La Crosse virus (LACV) (Family: *Bunyaviridae*, Genus: *Orthobunyavirus*, Serogroup: California) is the leading cause of arboviral neuroinvasive disease in children in the United States [1,2]. LACV encephalitis occurs primarily in the upper Midwestern and the Eastern United States, reflecting the distribution of the mosquito vector, *Aedes triseriatus *(Say), and its preferred vertebrate hosts, chipmunks and tree squirrels. LACV is transovarially transmitted by *Ae. triseriatus *and overwinters in the diapausing eggs [3-5].

In the laboratory, the transovarial transmission (TOT) rate (percentage of infected females that transmit virus to their progeny) and filial infection rate (FIR, percentage of infected progeny from a female) can each exceed 70% [6]. However, LACV infection rates in *Ae. triseriatus *collected as eggs or larvae from the field are much lower. For example, LACV was isolated from only 10 of 1,698 (infection rate = 0.006) mosquitoes that were collected as larvae from overwintered eggs [5]. In another study, the minimum field infection rates for LACV in larvae from overwintered eggs ranged from 0.003 - 0.006 [7]. The dramatic difference in LACV infection rates between field and laboratory studies could result from deleterious effects of virus infection on embryos during stressful periods, such as overwintering [8], or from virus clearance by the innate immune response of the vector [9-12].

Mathematical models developed to investigate parameters that condition transmission and persistence in nature of LACV [13] and Keystone virus (KEYV) (Family: *Bunyaviridae*, Genus: *Orthobunyavirus*, Serogroup: California) [14,15] suggested that the observed field infection rates for LACV are insufficient to maintain the virus in nature. For KEYV, the model suggests that the TOT rate must be at least 0.1 and there must be vertebrate-mediated amplification in order for KEYV to be maintained in nature. Infection rates detected in field collected larvae are significantly less than 0.1 [4,5,7]. Even when using infection rates obtained in the laboratory, the models suggest that LACV could not persist by TOT alone for more than a few generations [6,13]. Horizontal transmission would be necessary to complement TOT to maintain a "stable" LACV prevalence from year to year in the vector population. However, herd immunity in chipmunks and tree squirrels in forested areas can exceed 90%; thus most mosquito feedings would be on dead end hosts, interrupting horizontal amplification of the virus [13-15]. Alternate mechanisms must condition LACV persistence in its endemic foci.

LACV could be maintained in nature by stabilized infection of *Ae. triseriatus*. Stabilized infection was first observed with Sigma virus (SIGMAV, Family: *Rhabdoviridae*) and *Drosophila melanogaster *fruit flies [16]. Infection of female *D. melanogaster *with SIGMAV by inoculation resulted in a "nonstabilized" infection with a small proportion of the developing oocytes and the resultant progeny becoming transovarially infected [17]. However, if germarium infection occurred, the progeny were stably-infected, and SIGMAV was transmitted to nearly 100% of progeny. A relatively small number of stably-infected females could maintain virus prevalence at a constant level, assuming that any detrimental effects of the infection (e.g., longevity, fecundity, and development) are balanced by horizontal transmission [18,19]. Stabilized infection with California encephalitis virus (CEV) (Family: *Bunyaviridae*, Genus: *Orthobunyavirus*, Serogroup: California) has been demonstrated in *Ae. dorsalis *[19]. Stably-infected females transmitted the virus to more than 90% of progeny through five laboratory generations. Analysis of field collected *Ae. triseriatus *mosquitoes suggested the possibility of stabilized LACV infection [20]. Some *Ae. triseriatus *mosquitoes collected as eggs from the field and processed individually contained large amounts of LACV antigen and LACV RNA [20]. We designated these as super-infected (SI+) mosquitoes, and our current working hypothesis is that these SI+ mosquitoes represent stably-infected lineages of *Ae. triseriatus*.

To establish a stabilized infection in *Ae. triseriatus*, LACV must avoid or perturb the vector innate immune response. RNAi and apoptosis are potent anti-arboviral innate immune responses in mosquitoes [9-12,21,22]. Recent studies revealed the fundamental role of autophagy in *D. melanogaster *response to vesicular stomatitis virus infection [23,24]. Importantly, ovarian follicle degeneration in *D. melanogaster *is conditioned by both apoptosis and autophagy, which share some common signaling pathway caspase components [25,26]. Because of the critical role of TOT, it would be especially important for LACV to avoid induction of an autophagic response in infected follicles [27]. Some viruses that infect arthropods have evolved viral inhibitors of RNAi [28]. Tomato spotted wilt virus (Family: *Bunyaviridae*, Genus:*Tospovirus*), NSs protein suppresses RNA silencing in infected plants [29]. Arboviruses in the family *Bunyaviridae *can modulate the vertebrate host innate immune response. For example, the LACV NSs protein can counteract the RNAi response [30] and the Rift Valley fever virus (Family: *Bunyaviridae*, Genus: *Phlebovirus*) NSm protein can suppress apoptosis [31] in vertebrate cells. However, little is known about the role of these genes in perturbing vector innate immune responses. LACV induces an RNAi response in both *Aedes albopictus *and *Ae. triseriatus *mosquito cell cultures that is not suppressed by the NSs protein [32], but nothing is known about this response *in vivo *in tissues and organs of *Ae. triseriatus*.

The goals of this study were to investigate the prevalence of SI+ mosquitoes in sites in the LACV endemic region, to determine the genetic relatedness of the SI+ virus isolates, and to characterize LACV genes potentially associated with perturbation of apoptotic/autophagic and RNAi responses in SI+ mosquitoes.

## Results

### Detection of three LACV infection phenotypes in *Ae. triseriatus *mosquitoes from field collected eggs

**Figure 1:**
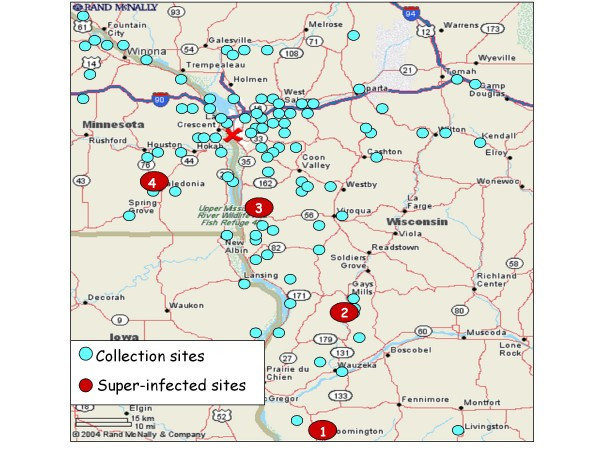
***Aedes triseriatus *mosquito collection sites in Minnesota, Wisconsin, and Iowa**. Circles represent the collection sites. Red circles are the sites where LACV super-infected mosquitoes were collected in 2006 and 2007. Site 1 - BEN2 Lafayette County, WI, Site 2 - NAT, Crawford County, WI. Site 3 - SVP Vernon County, WI, and Site 4 - CAL-GA Houston County, MN. La Crosse, WI is identified with the X.

Mosquitoes were collected as eggs from field sites in Wisconsin, Minnesota, and Iowa (Figure 1), hatched and reared to adults, and then assayed by immunofluorescence assay (IFA), virus isolation, and reverse transcription-PCR (RT-PCR). Based upon the results, mosquitoes were assigned to three infection phenotypes: SI+ mosquitoes contained infectious virus and major accumulations of viral antigen and nucleic acid, I+ mosquitoes contained detectable amounts of viral antigen and nucleic acid but no detectable virus in cell culture assays, and I- mosquitoes that contained no detectable viral antigen or nucleic acid or virus.

### IFA

**Figure 2:**
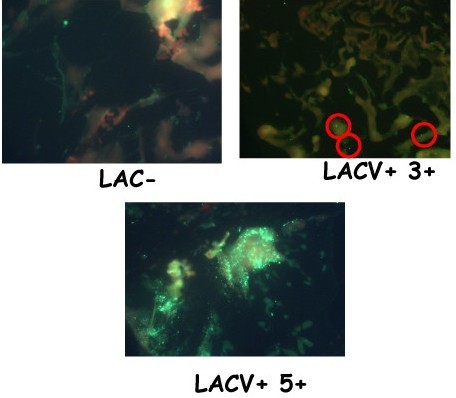
**La Crosse virus antigen in infected, field-collected *Aedes triseriatus *mosquitoes**. Mosquitoes were collected as eggs from the sites. Eggs were induced to hatch in the laboratory and emerged adults were assayed directly for the presence of LACV antigen by IFA (see Methods and Materials).

The three infection phenotypes were detected in field collections (Figure 2); the distribution and prevalence rates of the SI+ and I+ mosquitoes in collections made from the LACV endemic area in 2006 and 2007 are provided in Table 1. In total, 17,825 mosquitoes collected in 2006 and 2007 were assayed by IFA. Overall, 17 of 17,825 mosquitoes (prevalence rate: 0.0009) had the SI+ phenotype, 664 of 17,825 mosquitoes (prevalence rate: 0.037) were I+, and 17,161 of 17,825 mosquitoes were I- (Table 1). In 2006, 2 of 6,761 mosquitoes (prevalence rate = 0.0003) were SI+ compared to 15 of 11,064 mosquitoes (prevalence rate = 0.0014) in 2007.

**Table 1:** Prevalence and distribution of LACV SI+ and I+ mosquitoes in the 2006 and 2007 collections

	**I+**						**S+**					
	2006			2007			2006			2007		
	
County, State	PosMos	Total Tested	Prev	PosMos	Total Tested	Prev	Pos Mos	Total Tested	Prev	PosMos	Total Tested	Prev
Clayton, IA	N/A	N/A	N/A	3	198	0.015	N/A	N/A	N/A	0	198	0
Crawford, WI	12	555	0.022	68	1561	0.044	1	555	0.0018	4	1561	0.0026
Iowa, WI	22	792	0.028	4	188	0.021	0	792	0	0	188	0
LaCrosse, WI	12	605	0.02	129	3097	0.042	0	605	0	0	3097	0
Lafayette, WI	35	902	0.039	19	275	0.069	0	902	0	4	275	0.0145
Monroe, WI	1	183	0.006	42	1005	0.042	0	183	0	0	1005	0
Vernon, WI	37	1122	0.033	45	1770	0.025	1	1122	0.0009	0	1770	0
Houston, MN	89	1744	0.051	51	1318	0.039	0	1744	0	7	1318	0.0053
Winona, MN	24	858	0.028	74	1652	0.045	0	858	0	0	1652	0

Total	232	6761		432	11064		2	6761		15	11064	
												
Overall Prevalence**			0.034			0.039			0.0003			0.0014

*Prevalence was determined by dividing the number of positive mosquitoes by the number tested for each county

**Overall prevalence was determined by dividing the total number of positive mosquitoes by the total number of mosquitoes tested each year

### Virus isolation and titer

IFA positive mosquitoes were assayed for infectious virus in cell culture. LACV was isolated only from the SI+ mosquitoes (data not shown). The LACV titer of the abdomen of 11 SI+ mosquitoes from the 4 different collecting sites ranged from 2.7 - 4.7 log_10 _TCID_50_/ml (average = 3.2 log_10 _TCID_50_/ml). This did not differ significantly from the mean titer observed in the TOT-permissive laboratory colonized mosquitoes (3.9 log_10_TCID_50_/ml) (p > 0.05) [20].

In contrast to the SI+ mosquitoes, LACV was not isolated in Vero E6 or BHK-21 cells from any of 213 I+ mosquitoes assayed. To potentially increase virus isolation sensitivity, supernatant fluid homogenates of 22 I+ mosquitoes were blind passaged [33] in BHK-21 and Vero E6 cell monolayers and some were assayed by intrathoracic inoculation of *Ae. triseriatus *mosquitoes, but again no isolates were obtained (data not shown).

### RT-PCR

IFA positive mosquitoes were also processed by RT-PCR to amplify viral RNA sequences for phylogenetic, gene structure, and molecular evolution analyses. RNA was readily amplified from all SI+ mosquitoes. Viral RNA was also amplified from I+ mosquitoes, but the RT-PCR assay and protocols needed further optimization, presumably because of reduced amounts of viral RNA in some mosquitoes [34]. For later analyses, we developed a nested RT-PCR system to more easily amplify sequences from I+ mosquitoes.

### Distribution of LACV SI+ and I+ mosquitoes in geographic field collections of *Ae. triseriatus*

The prevalence of LACV-infected mosquitoes was determined in field collections from 2006 and 2007 (Table 1). In 2006, SI+ mosquitoes were collected from Vernon County (SVP/Vernon, WI/Mosquito/2006 Site 3 (Figure 1)) and Crawford County (NAT/Crawford, WI/Mosquito/2006 Site 2 (Figure 1)). In 2007, SI+ mosquitoes were collected from Crawford County (NAT/Crawford, WI/Mosquito/2007), Lafayette County (BEN2/Lafayette, WI/Mosquito/2007 Site 1 (Figure 1)) and Houston County (CAL-GA/Houston, MN/Mosquito/2007/Site 4 (Figure 1)) (Table 1). Prevalence rates of SI+ mosquitoes differed between sites and years, ranging from 0.0009 at one site in Vernon County, WI in 2006 to 0.015 at a site in Lafayette County, WI in 2007. Notably the prevalence rate for SI+ mosquitoes at one site in Crawford County, WI was 0.018 in 2006 and 0.026 in 2007. This was the only site to yield SI+ mosquitoes in both years of the study.

### Prevalence of SI+ mosquitoes in selected "hot spots"

To further investigate the prevalence of SI+ mosquitoes, eggs from multiple liners were hatched from "hot spots" where >1 SI+ mosquito had been detected previously. The SI+ and I+ prevalence rates were determined (Table 2). SI+ prevalence rates ranged from 0.012 to 0.121 in hot spots. At the SVP/Vernon, WI/2006 site, 1 of 84 (prevalence rate = 0.012) was SI+ (Table 2). At the BEN2/Layfayette, WI/2007 site, 4 of 220 (0.018) were SI+. At the CAL-GA/Houston, MN/2007 site, 7 of 58 mosquitoes (0.12) were SI+ (Table 2). Field collected mosquitoes from the respective sites were tested throughout the summer and interestingly, SI+ mosquitoes were only identified at each collection site once a year (Table 2).

**Table 2:** Prevalence of SI+ and I+ mosquitoes at "hot spots" in 2006 and 2007

Site/Year	County	Date	#Mosquitoes	I+ (Prevalence)	SI+ (Prevalence)
SVP/2006	Vernon, WI	8/31/2006	84	7 (0.083)*	1 (0.012)
NAT/2006	Crawford, WI	2006	67	5 (0.074)	1 (0.015)
NAT/2007	Crawford, WI	7/17/2007	475	30 (0.063)**	4 (0.008)
BEN 2/2007	Lafayette, WI	9/10/2007	220	15 (0.068)*	4 (0.018)
CAL-GA/2007	Houston, WI	8/27/2007	58	7 (0.121)**	7 (0.121)

Total			904	64 (0.071)	17 (0.019)

* p ≤ 0.05, ** p ≤ 0.01

I+ mosquitoes were also more prevalent in hot spots. The I+ prevalence rate for each site was compared to the overall prevalence rate of 0.038 using a Fisher's exact test to determine whether they differed significantly (Table 2). The prevalence of I+ mosquitoes in NAT/Crawford, WI/2007 and CAL-GA/Houston, MN/2007 differed significantly (p = 0.01) from the overall prevalence rate of I+ mosquitoes. The I+ prevalence rates observed in SVP/Vernon, WI/2006 and BEN2/Layfayette, WI/2007 were significantly greater (p = 0.05) than the overall I+ prevalence rate.

### Phylogenetic analysis of virus isolates from SI+ mosquitoes

**Figure 3:**
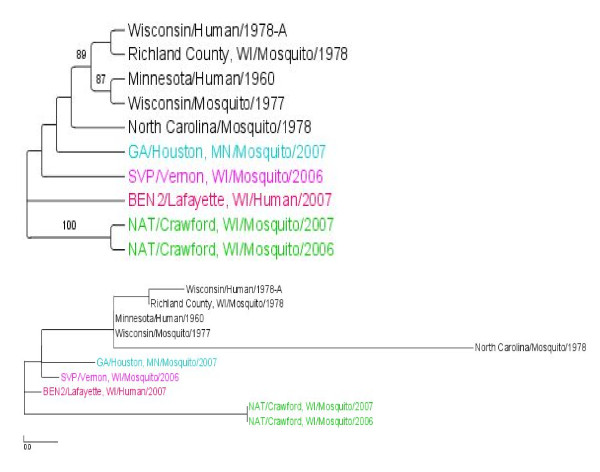
**Maximum Likelihood (ML) tree derived for the LACV S segment using RAxML**. The top figure is a cladogram; numbers over branches indicate % bootstrap support. The bottom figure is a phylogram. Length of scale bar = 0.01. Colored branches correspond to sites in Figure 1.

**Figure 4:**
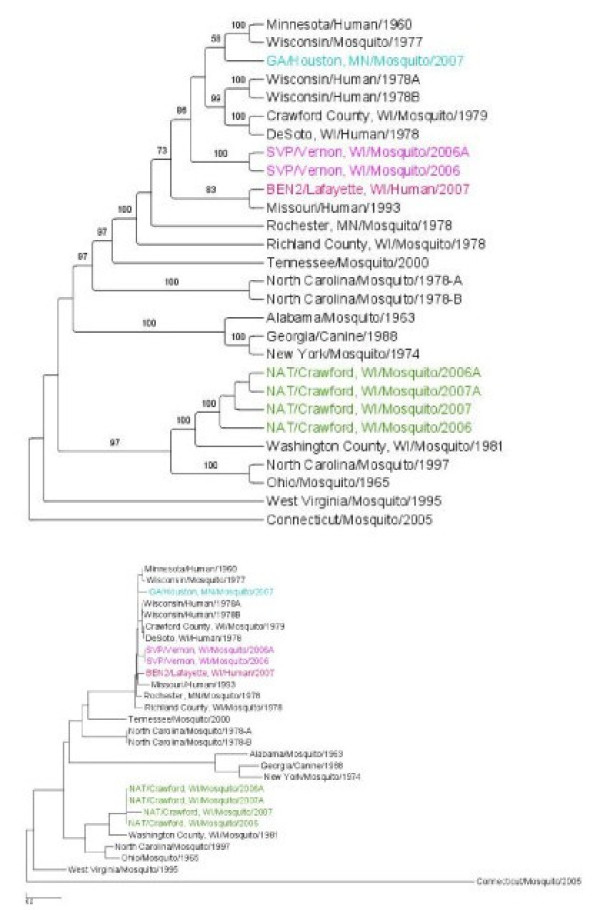
**Maximum Likelihood (ML) tree derived for the LACV M segment using RAxML**. The top figure is a cladogram; numbers over branches indicate % bootstrap support. The bottom figure is a phylogram. Length of scale bar = 0.01. Colored branches correspond to sites in Figure 1.

**Figure 5:**
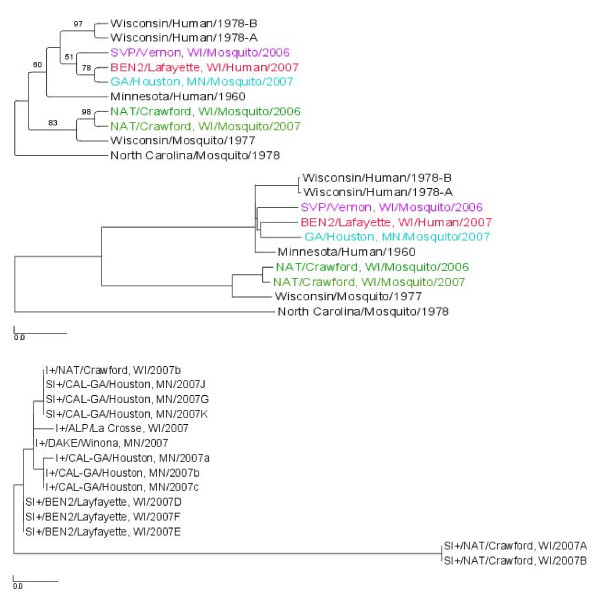
**Maximum Likelihood (ML) tree derived for the LACV L segment using RAxML**. The top figure is a cladogram; numbers over branches indicate % bootstrap support. The bottom figure is a phylogram. Length of scale bar = 0.01. Colored branches correspond to sites in Figure 1.

**Figure 6:**
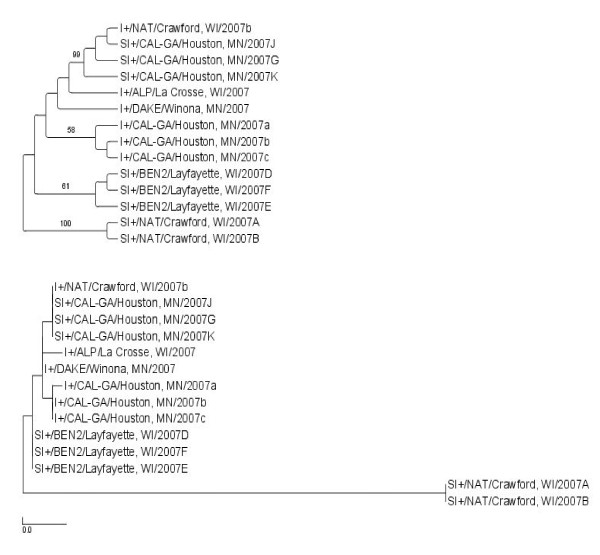
**Maximum Likelihood (ML) tree derived for the LACV NSm sequence of SI+ and I+ mosquitoes using RAxML**. The top figure is a cladogram; numbers over branches indicate % bootstrap support. The bottom figure is a phylogram. Length of scale bar = 0.01. Colored branches correspond to sites in Figure 1.

The three genome segments of plaque purified viruses from selected SI+ mosquitoes were sequenced and deposited in GenBank (Table 3). The sequences were analyzed phylogenetically alongside previously published LACV sequences (Table 3) using a maximum likelihood (ML) analysis to test whether SI+ isolates represent 1) a monophyletic group that is 2) phylogenetically distinct previously published LACV sequences. One virus isolate was analyzed from each of the four SI+ collection sites (Figure 1). ML trees were created for the entire S, M and L segments (Figures 3, 4, and 5, respectively) and for the NSm gene (Figure 6).

**Table 3:** Virus RNA sequences used in phylogenetic and molecular evolutionary analyses of virus isolates.

Virus Isolate^a^	Pheno-type	Passage History	Segment/Gene	Accession Number	Ref
Minnesota/Human/1960		C6/36 2	S	EF485030	[32]

			M	EF485031	

			L	EF485032	

Alabama/Mosquito/1963		Suckling mice 3	M	DQ426682	[33]

Ohio/Mosquito/1965		Suckling mice 4, Vero 1	M	DQ426683	[33]

New York/Mosquito/1974		Suckling mice 4, BHK 4	M	D10370	[34]

Wisconsin/Mosquito/1977		Unknown	S	DQ1961120	[32]

			M	DQ196119	

			L	DQ196118	

Wisconsin/Human/1978-A		Mouse brain 1, BHK2, Vero 1	S	EF485033	[32]

			M	EF485034	

			L	EF485035	

Wisconsin/Human/1978-B		Mouse brain 1, BHK2	S	NC004110^b^	

			M	NC004109^b^	

			L	NC004108^b^	

Rochester, MN/Mosquito/1978		Unknown	M	DQ426680	[33]

DeSoto, WI/Human/1978		Suckling mice 2, BHK 2	M	U18980	[35]

Richland County, WI/Mosquito/1978		Suckling mice 2, BHK 1	M	U70206	[36]

North Carolina/Mosquito/1978-A		Mouse brain 1, Vero 3	S	EF485036	[33]

			M	EF485037	

			L	EF485038	

North Carolina/Mosquito/1978-B		Suckling mice 2, Vero 2	M	DQ426681	[33]

Crawford County, WI/Mosquito/1979		Suckling mice 2, BHK 1	M	U70207	[36]

Washington County, WI/Mosquito/1981		Suckling mice 2, BHK 1	M	U70208	[36]

Georgia/Canine/1988		Veros 1, Suckling mice 1	M	DQ426684	[33]

Missouri/Human/1993		Vero 1	M	U70205	[36]

West Virginia/Mosquito/1995		Vero 1	M	DQ426685	[33]

North Carolina/Mosquito/1997		Vero 1	M	DQ426686	[33]

Tennessee/Mosquito/2000		Vero 1	M	DQ426687	[33]

Connecticut/Mosquito/2005		Vero 1	M	DQ426688	[33]

SVP/Vernon, WI/Mosquito/2006		Vero 2	S	GU596389	

			M	GU596384	

			L	GU596378	

NAT/Crawford, WI/Mosquito/2006		Vero 2	S	GU596387	

			M	GU596382	

			L	GU596379	

NAT/Crawford, WI/Mosquito/2007		Vero 2	S	GU596388	

			M	GU596383	

			L	GU596377	

BEN2/Lafayette, WI/Mosquito/2007		Vero 2	S	GU596386	

			M	GU596381	

			L	GU596376	

CAL-GA/Houston, MN/Mosquito/2007		Vero 2	S	GU596390	

			M	GU596385	

			L	GU596380	

LAC01/SVP/LaCrosse, WI/Mosquito/2006A	SI+	Vero 1	NSs	GU564182	

			NSm	N/A	

LAC03/NAT/Crawford, WI/Mosquito/2007A	SI+	Vero 1	NSs	GU564183	

			NSm	GU564201	

LAC05/KBT/Monroe, WI/Mosquito/2007A	I+	None	NSs	GU564184	

			NSm	GU564202	

LAC06/GOLF/Houston, MN/Mosquito/2007A	I+	None	NSs	GU564185	

			NSm	GU564203	

LAC07/HIDV/Winona, MN/Mosquito/2007A	I+	None	NSs	GU564186	

			NSm	GU564204	

LAC08/NAT/Crawford, WI/Mosquito/2007B	I+	None	NSs	GU564187	

			NSm	GU564205	

LAC09/HIDV/Winona, MN/Mosquito/2007B	I+	None	NSs	GU564188	

			NSm	GU564206	

LAC10/NAT/Crawford, WI/Mosquito/2007C	I+	None	NSs	GU564189	

			NSm	GU564207	

LAC11/NAT/Crawford, WI/Mosquito/2007D	I+	None	NSs	GU564190	

			NSm	N/A	

LAC12/LCVP/Houston, MN/Mosquito/2007A	I+	None	NSs	GU564191	

			NSm	GU564208	

LAC13/ALP/LaCrosse, WI/Mosquito/2007A	I+	None	NSs	GU564192	

			NSm	GU564209	

LAC14/DAKE/Winona, MN/Mosquito/2007A	I+	None	NSs	GU564193	

			NSm	GU564210	

LAC16/NAT/Crawford, WI/Mosquito/2007B	SI+	None	NSs	GU564194	

			NSm	GU564211	

LAC19/BEN2/Lafayette, WI/Mosquito/2007D	SI+	None	NSs	GU564195	

			NSm	GU564212	

LAC20/BEN2/Lafayette, WI/Mosquito/2007E	SI+	None	NSs	GU564196	

			NSm	GU564213	

LAC21/BEN2/Lafayette, WI/Mosquito/2007F	SI+	None	NSs	GU564197	

			NSm	GU564214	

LAC22/CAL-GA/Houston, MN/Mosquito/2007G	SI+	Vero 1	NSs	GU564198	

			NSm	GU564215	

LAC23/CAL-GA/Houston, MN/Mosquito/2007J	SI+	Vero 1	NSs	GU564199	

			NSm	GU564216	

LAC24/CAL-GA/Houston, MN/Mosquito/2007K	SI+	Vero 1	NSs	GU564200	

			NSm	GU564217	

LAC27/CAL-GA/Houston, MN/Mosquito	I+	None	NSm	GU564218	

LAC28/CAL-GA/Houston, MN/Mosquito	I+	None	NSm	GU564219	

LAC29/CAL-GA/Houston, MN/Mosquitor	I+	None	NSm	GU564220	

^a ^All viruses isolated from mosquitoes were isolated from *Ae. triseriatus *except for AL/Mosquito/1963 (*Psorophora howardii*) and TN/Mosquito/2000 (*Ae. albopictus*)

^b ^Previous submission by Hughes *et al*. 2002.

The S segment ML tree (Figure 3) contained 3 well supported clades. One clade contained virus isolates from Wisconsin and Minnesota with 89% support. Internal to this, a clade with 87% support contained the MN Human 1960 and the WI Mosquito 1977 isolates. The third clade with 100% support contained the NAT/Crawford, WI/2006 and 2007 SI+ isolates. Note the long branch associated with NAT/Crawford clade demonstrating that these SI+ isolates are genetically distinct. However, also note that these two isolates collected from the same site in consecutive years are very similar to one another. The other three SI+ isolates are paraphyletic and therefore not phylogenetically distinct from the previously published LACV isolates. The S segment therefore suggests that SI+ isolates are not a monophyletic group. The trees in Figure 3 are not rooted and therefore do not suggest that SI+ lineages are basal.

The M segment ML tree (Figure 4) contains many well supported clades. As with the S segment, M segments in SI+ isolates do not form a monophyletic group and instead arise four times independently on clades that share a common ancestor with the previously published LACV isolates. Further, unlike the S phylogeny, in no case are SI+ isolate branch lengths long, suggesting sequence similarity to the previously published isolates. Note again that the NAT/Crawford isolates are very similar to one another.

The L segment ML tree contained the same patterns found in the M segment ML tree (Figure 5). One interesting additional observation is the phylogenetic placement of the isolate Wisconsin/Mosquito/1977. In the S segment analysis, it is in a clade with all the LACV isolates from Wisconsin and Minnesota, whereas in the L segment analysis the isolate is in a clade with the isolates from NAT/Crawford, WI/2006 and/2007. This is likely evidence for segment reassortment.

### Phylogenetic analysis of the NSs and NSm genes from SI+ and I+ mosquitoes

The multiple functions of the NSs (S segment) and NSm (M segment) proteins in the viral life cycle make these proteins strong candidates for conditioning the I+ and SI+ phenotypes. To examine this hypothesis, LACV NSs and NSm genes from 8-12 mosquitoes of both phenotypes were sequenced and compared in an effort to correlate the SI+/I+ phenotypes with nucleotide and/or amino acid differences. NSm genes were also sequenced from SI+ and I+ mosquitoes collected from the same locations in the same year. NSs and NSm nucleotide sequences were deposited in GenBank (Table 3).

NSs sequences were determined for 10 I+ amplicons and nine SI+ isolates collected in 2006 and 2007. No NSs nucleotide differences were observed among the nineteen isolates (Additional File - Figure 1). This analysis was repeated with the NSm gene and the same phylogenetic patterns as described for the S, M, and L segments were noted (Figure 6). SI+ isolates are polyphyletic. Note that despite the similarity of the M segments from NAT/Crawford SI+ isolates (Figure 4), these isolates are very genetically distinct from all other isolates.

### Molecular evolution of LACV isolates and NSm sequences

The number of segregating sites (S), unique haplotypes (Hap), and singletons η_e _for each gene in SI+ and previously published LACV isolates and between NSm sequences from I+ and SI+ mosquitoes are listed in Table 4. Also listed are overall nucleotide diversity (π), π_s _among synonymous sites and π_a _among replacement sites in the 6 protein encoding genes. Theta from pair-wise comparisons and F* are also listed.

**Table 4:** Molecular evolution rates^a ^among LACV isolates and NSm genes from SI+ and I+ mosquitoes.

Domain	Sites	S	Hap	η_ε_	π	π_σ_	π_α_	π_α_/π_σ_	θ	F*
S segment										
SI+ isolates										
5' S non-coding (nt 1-81)	81	2	3	0	0.0124				0.96	0.2386
Nucleocapsid (nt 82-789)	708	11	4	2	0.0088	0.0378	0.0000	-	5.28	1.3261
NSs (nt 102-379)	278	0	1	1	0.0000	0.0000	0.0000	-	0.01	-
3' S non-coding (nt 787-984)	195	5	4	2	0.0133				2.40	0.5779

All	984	18	11	**5**	0.0100				8.64	1.0574
Previous isolates										
5'		1	2	1	0.0059				0.48	-0.7715
Nucleocapsid		13	3	11	0.0088	0.0378	0.0000	-	6.24	-0.7910
NSs		1	2	0	0.0017	0.0108	0.0000	-	0.48	1.1573
3'		9	3	9	0.0222				4.32	-1.2451

All		23	4	**21**	0.0098				11.04	-1.0400

M segment										
SI+ isolates										
5' M non-coding (nt 1 - 61)	61	1	2	0	0.0094				0.41	1.1015
G2 (nt 62-961)	900	44	4	3	0.0267	0.1007	0.0041	0.0411	17.96	1.7100*
NSm (nt 962-1483)	522	37	4	1	0.0400	0.1626	0.0056	0.0344	15.10	1.8884**
G1(nt 1484-4388)	2905	190	7	25	0.0346	0.1406	0.0041	0.0292	77.55	1.4902
3' M non-coding (nt 4389-4526)	138	17	6	7	0.0549				6.94	0.3115

Total	4526	289	7	**36**	0.0339				117.96	1.5238
Previous Isolates										
5'		3	3	3	0.0089				1.02	-2.0309
G2		104	8	79	0.0287	0.1040	0.0060	0.0574	36.87	-1.7512
NSm		80	8	57	0.0373	0.1303	0.0111	0.0851	28.00	-1.6129
G1		421	9	296	0.0361	0.1378	0.0068	0.0492	145.79	-1.5991
3'		14	5	13	0.0225				5.12	-2.1718

		622	9	**448**	0.0340				216.80	-1.6626

L segment										
SI+ isolates										
5' L non-coding (nt 1-61)	61	2	3	0	0.0197				0.96	1.4316
RNA polymerase (nt 5572-12360)	6789	249	5	79	0.0197	0.0878	0.0016	0.0179	119.52	0.9799
3' non-coding (nt 12361-12490)	131	6	4	3	0.0260				3.36	0.4325

Total	6980	257	5	**82**	0.0198				123.84	0.9754
Previous isolates										
5'		4	4	3	0.0295				1.92	-0.4175
RNA polymerase		398	5	288	0.0270	0.1204	0.0021	0.0177	192.96	-0.4076
3'		6	5	2	0.0244				2.88	0.7913

Total		408	5	**293**	0.0270				197.76	-0.3893
NSm gene										
SI+ mosquitoes	522	37	3	0	0.0308	0.1257	0.0042	0.0334	16.0710	1.5685*
I+ mosquitoes		5	5	4	0.0037	0.0140	0.0008	0.0587	1.9330	-0.8121

^a ^Sites = number of nucleotides in each gene or segment region. S = number of segregating sites. Hap = number of distinct haplotypes. η_e _= number of singletons, π = nucleotide diversity, the average number of nucleotide differences per site between two sequences, π_s _= nucleotide diversity among synonymous sites, π_s _= nucleotide diversity among replacement sites, θ = E(π expected number of pairwise differences in S, F* = Fu and Li (1993). * |F*| > 0 with probability of 0.05, ** |F*| > 0 with probability of 0.01

**Figure 7:**
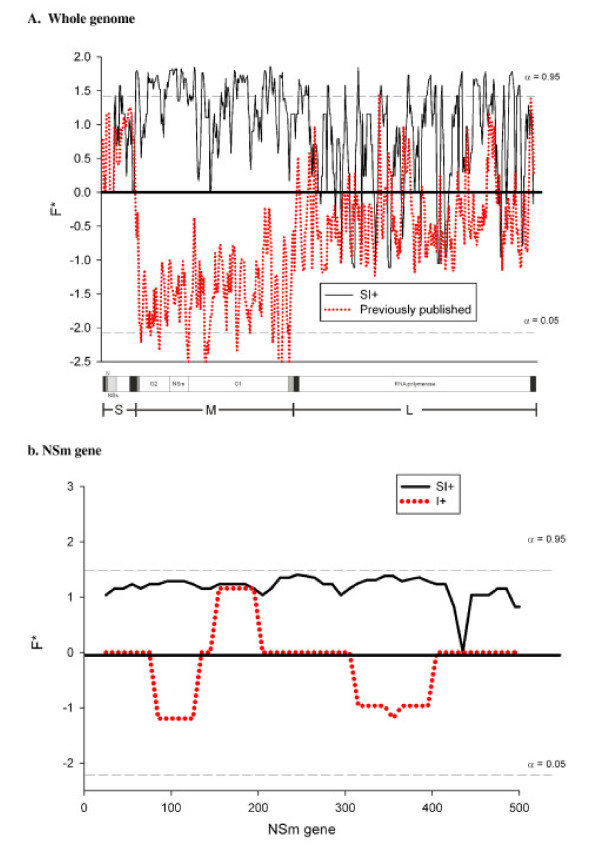
**Molecular evolution of LACV: Comparison of segregating sites, haplotypes, and singletons between the three RNA segments of SI+ isolates and previously published isolates (7a) and between NSm genes from I+ and SI+ mosquitoes (7b)**.

Table 4 indicates five patterns. First, the numbers of segregating sites are consistently larger in previously published isolates as compared to SI+ isolates (1.3, 2.2 and 1.6 fold higher in the S, M, and L segments, respectively). The only exception was seen in the NSm gene where there were 37 segregating sites among SI+ isolates as compared with five sites in previously published isolates. Similarly, the expected numbers of segregating sites (θ) are consistently larger in previously published isolates (1.3, 1.8 and 1.6 fold higher in the S, M, and L segments respectively) but 8 fold higher in the NSm gene in SI+ as compared with previously published isolates. Second, the numbers of singletons were much larger in previously published isolates (4.2, 12.4 and 3.6 fold higher in the S, M, and L segments, respectively) as compared with the SI+ isolates. This trend was especially pronounced in the NSm gene in SI+ isolates. Despite having 37 segregating sites, none of these were singletons in SI+ but four of the five sites were singletons in previously published isolates. Third, the three measures of nucleotide diversity (π) did not differ greatly between previously published and SI+ isolates. Diversity was greatest in the M segment and least in the S segment. The only exception was again seen in the NSm gene where there was 9 fold greater π among SI+ isolates as compared with I+ isolates. Fourth, the ratio π_a_/π_s _was small in all comparisons indicating that the majority of mutations are synonymous and the rate of amino acid evolution is slow. Fifth, and most noteworthy, F* was consistently positive among SI+ isolates and consistently negative among previously published isolates in all three segments and in the NSm gene. However, this difference was most pronounced (and significant) in the M segment, particularly in the G2 and NSm genes. This difference is most obvious when F* is plotted separately for SI+ and previously published isolates across the entire genome (Figure 7a) and between SI+ and I+ in the NSm gene (Figure 7b).

Relative Synonymous Codon Usage (RSCU) was analyzed within and among I+ and SI+ isolates using the GCUA (General Codon Usage Analysis) package [35]. Clusters as revealed by correspondence analysis, principal components analysis and cluster based upon McInerney's distance measure completely overlapped between SI+ and previously published isolates indicating no general differential RSCU between the groups.

### Nucleotide differences between LACV SI+ and previously published isolates and between LACV NSm sequences from I+ and SI+ mosquitoes

**Figure 8:**
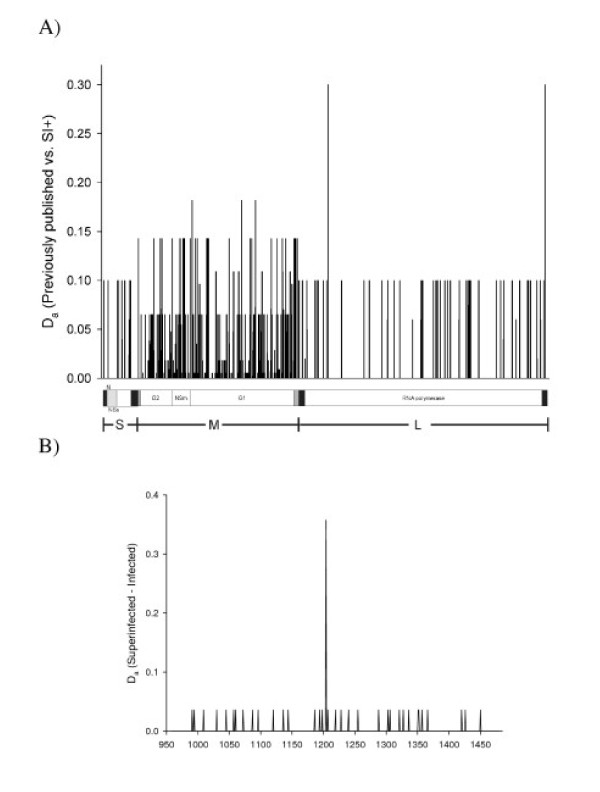
**Nucleotide differences (D_a_) between RNA segments from LACV SI+ and previously published isolates (a) and between NSM sequences from SI+ and I+ mosquitoes (b)**.

The number of net nucleotide substitution differences/site (D_a _= D_I+/SI+ _- ((D_I+ _+ D_SI+_)/2)) [36]) between LACV isolates from SI+ and previously published LACV sequences were plotted for each site in the three segments (Figure 8a), and for the NSm gene from SI+ and I+ mosquitoes (Figure 8b) to test for large nucleotide differences. Figure 8a clearly indicates that the majority of differences arise on the M segment. Table 5 lists the nucleotides at each site with a D_a _> 0.1. These appear as nucleotides in non-coding regions or as codons in the protein coding regions. The RSCU for the LACV isolates are listed for each substitution. RSCU values that differed by > 0.1 between the SI+ and previously published isolates are grey highlighted. The average RSCU for SI+ isolates and previously published isolates appear at the bottom of the table. All of the substitutions with D_a _> 0.1 were synonymous. A pair-wise t-test of differences in RSCU between SI+ and previously published isolates was performed and was not significant.

**Table 5:** Substitutions that differ in frequency between SI+ and previous isolates with Da > 0.10.

Genome			SI+	**Prev. publ**.	RSCU	RSCU
Region		Da	codon	Codon	SI+	**Prev. publ**.
M segment						
5' noncoding						
42	5'NC	0.143	A	G	-	-
G2						
478	Val	0.143	GUA	GUG	0.254	0.210
658	Gln	0.143	CAA	CAG	0.540	0.460
697	Leu	0.143	UUG	UUA	0.233	0.300
NSm						
991	Ile	0.143	AUU	AUA	0.287	**0.544**
1204	Leu	-0.027 - 0.357	CUA	CUU	0.177	0.116
1207	Phe	0.143	UUC	UUU	0.453	0.547
1288	Leu	0.143	UUA	UUG	0.300	0.233
1306	Phe	0.143	UUU	UUC	0.547	0.453
1327	Cys	0.143	UGU	UGC	0.472	0.528
G1						
1501	Thr	0.143	ACU	ACC	**0.316**	0.173
1543	Pro	0.182	CCG	CCA	0.054	**0.492**
1639	Leu	0.143	CUG	CUA	0.114	0.177
1690	Arg	0.143	AGG	AGA	0.422	**0.578**
1951	Gly	0.143	GGA	GGG	**0.288**	0.114
1984	Leu	0.143	CUG	CUA	0.114	0.177
2002	His	0.143	CAC	CAU	0.354	**0.646**
2584	Gly	0.143	GGA	GGG	0.288	0.274
2929	Pro	0.182	CCA	CCG	**0.492**	0.054
3169	Lys	0.143	AAG	AAA	0.381	**0.619**
3214	His	0.143	CAC	CAU	0.354	**0.646**
3310	Gly	0.182	GGA	GGG	0.288	0.274
3754	Val	0.143	GUA	GUG	0.254	0.210
3916	Leu	0.143	UUA	UUG	0.300	0.233
4066	Asn	0.143	AAC	AAU	0.307	**0.693**
4156	Lys	0.143	AAG	AAA	0.381	**0.619**
3' noncoding						
4395	3'NC	0.143	G	A	-	-
4430	3'NC	0.143	C	U	-	-
4482	3'NC	0.143	G	A	-	-
L segment						
817	Ser	0.300	UCA	UCG	**0.269**	0.048
3' noncoding						
6888	3'NC	0.300	U	C	-	-

Average					0.317	0.362
T-test [25 d.f.] prob.					0.130	

All codon substitutions are synonymous. The codon used and the associated RSCU are listed for each substitution. Grey highlighted values differed by > 0.1 between the previously published and SI+ isolates. The average RSCU for previously published and SI+ isolates appears at the bottom of the table. The probability value is from a pair-wise t-test of differences in RSCU between SI+ and previously published isolates.

In the overall analysis, a single large difference was detected in NSm gene sequences from SI+ and I+ mosquitoes. The QTN identified in position 246 of the NSm gene (Figure 8b) corresponds to a U to A transversion in the third position of a CUN Leu codon. This codon shows severe bias towards CUU (57 codons in 14 mosquitoes) compared to a CUA (7 codons in 14 mosquitoes). All 7 CUA codons appeared in SI+ mosquitoes but none appeared in the I+ mosquitoes. The difference was significant using Fisher's exact test (P-value = 0.01437).

### LACV NSm nucleotide and amino acid differences between LACV SI+ and I+ mosquitoes from one site

**Figure 9:**
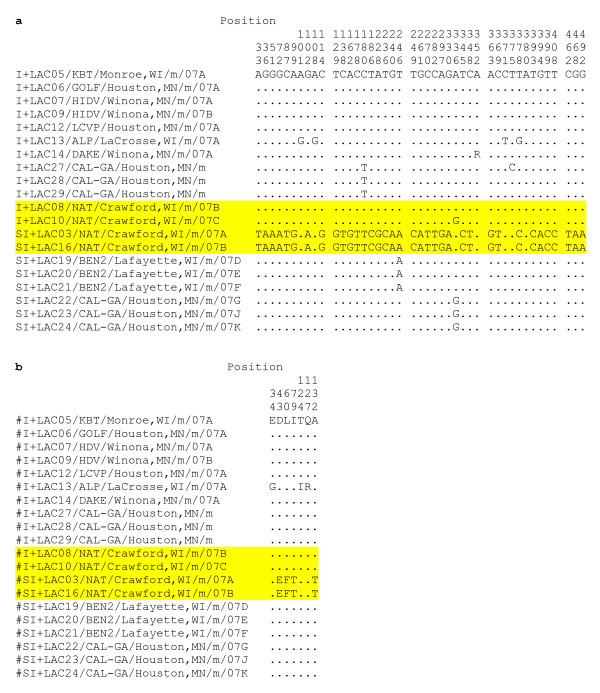
**LACV NSm NT (a) and AA (b) differences between SI+ and I+ mosquitoes from one site**. Sequences from the NAT site are highlighted in yellow.

Two SI+ and two I+ mosquitoes, respectively, were detected at the NAT site (Figure 9). The SI+ and I+ sequences differed in numerous nts (Figure 9a). Four LACV NSm amino acids differentiated the two SI+ mosquitoes (NAT/Crawford, WI, NAT/2007A and NAT/2007B) from the two I+ mosquitoes (I+/NAT/Crawford, WI/2008a) and I+/NAT/Crawford, WI/2008b) (Figure 9b). These four amino acid changes: D43E, L60F, I79T, and A132T provide possible molecular correlates of I+ vs. SI+ phenotype. In contrast, the LACV NSs sequences of the four mosquitoes were absolutely conserved (Additional Files - Figure 1).

## Discussion

The detection of SI+ in addition to I+ mosquitoes (Figure 2) was surprising. I+ mosquitoes were only detected because we assayed individual mosquitoes by IFA and PCR. They did not yield infectious virus and would not be detected by cell culture bioassay [33]. I+ mosquitoes are >10-fold more common in nature than SI+ mosquitoes, and thus could pose a significant risk to humans (Table 1). When first detected, we thought I+ mosquitoes might be a laboratory artifact resulting from less than optimal collecting and processing conditions, which could have reduced virus stability, infectivity, and titer, resulting in the I+ phenotype. However, the finding was replicated in multiple years. I+ mosquitoes may have low titer infections in certain organs and tissues that could be rescued by environmental and physiological stimuli. For example, isolation of St. Louis encephalitis and West Nile viruses from overwintering mosquitoes [37,38] is enhanced when field collected *Culex pipiens *mosquitoes are held at ambient insectary temperatures and provided sugar meals before virus assay. However, I+ mosquitoes were hatched and reared to adults in the insectary before virus assay, which would have provided ample time and metabolic and cellular activity for LACV to replicate to detectable titer. Perhaps additional physiological stimuli (e.g., mating, oogenesis, gonadotrophic cycles) would rescue infectious virus from I+ mosquitoes. The sensitivity of virus isolation by cell culture bioassay may also be a confounding factor. In early studies, virus was isolated by inoculation of samples into suckling mice [5,7], which is more sensitive than virus isolation in cell culture [33]. However, the equally sensitive method of intrathoracic inoculation of *Ae. triseriatus *mosquitoes also failed to yield infectious virus (data not shown). These observations suggest that I+ mosquitoes represent non-productive or abortive infections of the mosquito. The I+ mosquitoes must be controlling or clearing the infection. If so, they could yield critical and fundamental information concerning the molecular, immunologic, and physiological bases of vector competence.

The SI+ mosquitoes are also of great interest. If they are stably-infected, a relatively small number of these females could maintain LACV in nature with a low general field infection rate [18]. SI+ mosquitoes were detected in the population at a low level (prevalence rate = 0.0009) (Table 1) but were widely distributed in the collection area (Figure 1). The prevalence of SI+ mosquitoes ranged from 0.0084 (NAT/Crawford County, WI/2007) to 0.12 (CAL-GA/Houston, MN/2007) (Table 2). The identification of SI+ mosquitoes in the same NAT site in two different years suggests that LACV is stabilized in mosquitoes in this collection site. Stabilized infection of *D. melanogaster *with SIGMAV occurs when the germarium of females become infected [16,17], resulting in virus TOT to nearly 100% of progeny. A relatively low number of stably-infected females can maintain SIGMAV at a low prevalence indefinitely in nature [39,40]. Studies are needed to determine if LACV infects the germarium of SI+ mosquitoes and is passed to all or most progeny in ensuing generations.

This also raises the question of whether individual mosquito families or populations are responsible for the SI+ phenotype or whether some LACV quasispecies arise by accident or at random in particular populations, and cause SI+ infections in mosquitoes in geographic islands. There is significant gene flow in *Ae. triseriatus *populations [41] including in our study area in the LaCrosse region [42]. Nonetheless, it is possible that in geographic islands an innate immune arms race could emerge between the virus and the vector resulting in stabilized infection.

Our molecular evolutionary analyses strongly argue that SI+ quasispecies evolve at random in particular breeding sites or geographic islands. SI+ isolates are phylogenetically similar but are polyphyletic. Synonymous substitution rates greatly exceeded replacement rates in all genes and isolates. Overall, no general differences in RSCU were detected between previously published and SI+ isolates. However, the overall comparison of the LACV NSm gene from I+ and SI+ mosquitoes did identify a single QTN (U ↔ A transversion) in a Leu (CUN) codon in the NSm gene (Figure 8b). The significance of this QTN remains to be determined.

A consistent and significant trend was detected by Fu and Li's F* analysis of singleton versus shared mutations (Figure 8a) and in direct comparison of I+ and SI+ mosquitoes (Figure 8b). S and L segments evolved in a manner consistent with purifying selection and subsequent neutralism. But the SI+ M segment had a large and significant excess of intermediate-frequency alleles while the M segment had a large, significant excess of singletons in previously published LACV sequences and in sequences from I+ mosquitoes. It is difficult to make any definite conclusions from the previously published sequences, which were collected over a period of 45 years and did not arise from an intensive spatial or temporal field sample. Thus, the chances of finding intermediate frequency η_i _alleles is small. Nevertheless, Figures 7a and 8a illustrate that most of the evolution of the past 45 years has occurred in the M segment.

The patterns in direct comparison of NSm sequences from I+ and SI+ mosquitoes in Figures 7b and 8b are consistent with an hypothesis that the error prone RNA-dependent RNA polymerase generates a constellation of genotypes and the majority of these generate I+ phenotypes in their mosquito host. The large accumulation of singletons in I+ isolates in third codon positions reflect the activity of error prone RNA-dependent RNA polymerase, filtered by purifying selection. In contrast, occasionally a SI+ genotype arises and, because they survive and are maintained at a higher rate in stably-infected mosquito lineages than I+ genotype, SI+ maintain an excess of intermediate-frequency η_i _alleles. These intermediate-frequency alleles may confer reduced detection and/or destruction via the RNAi or apoptotic/autophagic pathways. Disruptive selection would then increase to moderate frequencies those genotypes that avoided destruction in the mosquito host and become manifested as SI+ mosquitoes. A much larger proportion of novel gene sequences in the M segment would yield viable virus but which would only partially avoid detection and/or destruction. Selection would not increase the frequencies of these genotypes and instead they would be manifested as a large frequency of singleton η_e _alleles. This trend was especially pronounced in the M segment in general and in the NSm gene in SI+ isolates in particular. There was >9 fold nucleotide diversity in the NSm gene among SI+ isolates as compared with I+ isolates (Table 4). Despite having 37 segregating sites, none of these were singletons in SI+ while four of only five sites were singletons in I+ individuals. This suggests that disruptive selection was increasing the frequency of shared (η_i_) genotypes in SI+ mosquitoes while no such selection favored any of the I+ genotypes resulting in singletons (η_e_) in those mosquitoes. Overall, LACV NSm sequences in SI+ mosquitoes differed between sites, suggesting that different polymorphisms in NSm may condition the SI+ phenotype. These polymorphisms would be expected in a quasispecies model of LACV infection in mosquitoes, and different polymorphisms in NSm and *Ae. triseriatus *innate immune genes could condition the same SI+ phenotype in different sites or geographic islands in the endemic area.

In one site (NAT), we were able to characterize NSs and NSm gene sequences from SI+ and I+ mosquitoes (Figure 9). There were no differences in NT sequence in the NSs genes (Additional File - Figure 1), but there were multiple NT and four amino acid differences between LACV NSm gene from SI+ and I+ mosquitoes in the site (Figure 9). The role that these changes may have in conditioning the establishment of the respective phenotypes remains to be determined. Sequence analysis of the *Ae. triseriatus *inhibitor of apoptosis-1 (*AtIAP1*) gene in mosquitoes from our study area revealed extensive polymorphisms (approximately 3 fold greater diversity than in a typical mosquito gene). One would assume that this gene would be highly conserved to prevent apoptosis [43].

In the *D. melanogaster*-SIGMAV system, the *ref(2)P *gene, which determines stabilized virus infection of the host, is also highly variable, presumably as a result of the host counteracting genetic changes in the virus [44]. The fly *ref(2)P *locus has two principal alleles, *ref(2)P*^P ^and *ref(2)P*^O^, respectively, which determine whether or not the fly will restrict Sigma virus infection or be permissive to stabilized infection [45]. There is a well characterized innate immune arms race between the *ref(2)p *gene and SIGMAV N protein epitopes [40], which determines productive infection of the host, TOT of the virus, and stabilized infection. The gene product of the *ref(2)P *locus is a protein kinase in the Toll innate immune pathway [46]. Perhaps a similar major innate immune gene-for-gene interaction conditions the LACV SI+ and I+ phenotypes in *Ae. triseriatus*. The accumulating evidence suggests that interactions between the LACV NSm and the *AtIAP1 *gene may somehow condition the SI+ phenotype. In this regard, apoptosis and autophagy have been show to share common caspase regulatory pathway components, autophagy and apoptosis both occur during degeneration of ovarian follicles, and autophagy has just been recognized as a key antiviral response in *D. melanogaster *[23-26]. All of this is especially provocative in the context of the LACV-*Ae. triseriatus *system in which virus amplification and maintenance in nature is predicated upon productive infection of ovarian follicles. Infection of *Ae. triseriatus *ovarian follicles with West Nile virus, but not LACV, induces an autophagosomic response in the follicles (BJB, unpublished data). Reverse genetics capability is now available for LACV, and we can exploit this robust approach to investigate and identify potential viral determinants of the SI+ and I+ phenotypes [47].

Mating efficiency studies with the newly discovered SI+ and I+ mosquitoes are also needed. Previous studies demonstrated a fitness advantage with increased mating efficiency for LACV-infected mosquitoes from the field, but the mosquitoes were not phenotyped in terms of SI+ or I+ infection [48]. The mating advantage may be more pronounced in SI+ mosquitoes. Mathematical models of LACV and KEYV [13-15] need to consider such factors as stabilized infection or mating advantages resulting from such infection. These two factors may help maintain stable LACV prevalence from year to year in the vector population.

## Conclusions

Processing of individual adult *Ae. triseriatus *mosquitoes emerged from field collected eggs revealed the presence SI+ and I+ mosquitoes in nature. SI+ mosquitoes could represent stabilized infections. Such mosquitoes could be present in low numbers, but could nonetheless maintain the virus in an area. Understanding the molecular basis of the SI+ phenotype would provide biomarkers for improved risk assessment and for targeting control programs for LACV encephalitis and other arboviral diseases.

## Methods

### Egg collection

*Aedes triseriatus *eggs were collected by the La Crosse County Public Health Department from five oviposition traps in each of 119 sites in Minnesota (n = 30), Wisconsin (n = 83) and Iowa (n = 6) (Figure 1). Mosquito eggs were collected between mid-June and October of 2006 and 2007 in Crawford (2006: 8 sites, 2007: 15 sites), La Crosse (2006: 6 sites, 2007: 37 sites), Monroe (2006: 3 sites, 2007: 12 sites), Vernon (2006: 11 sites, 2007: 12 sites), Lafayette (2006 and 2007: 2 sites), and Iowa (2006: 2 sites, 2007: 4 sites) counties in Wisconsin, Winona (2006: 7 sites, 2007: 13 sites) and Houston (2006: 10 sites, 2007: 17 sites) counties in Minnesota and Clayton county (2007: 6 sites) in Iowa. The eggs were transported to the insectaries at the Arthropod-borne and Infectious Diseases Laboratory (AIDL) at Colorado State University (CSU), Fort Collins, CO and maintained in the insectary until hatching and processing.

### Immunofluorescence assay

Approximately 2 - 4 days post-emergence, a mosquito leg was removed, squashed onto an acid-washed microscope slide, and fixed in cold acetone. Legs were then assayed for LACV antigen by direct immunofluorescence assay (IFA) [5]. Variation in the amount of LACV antigen was evident among these mosquitoes. The degree of fluorescence was assigned quantitative scores to provide more information about the nature of the infection in the respective mosquitoes. An IFA score of 0 indicated no detectable antigen; 1 - small amounts of antigen in some tissues; 2 - small amounts of antigen in most tissues, 3 - significant amounts of antigen in some tissues, 4 - significant amounts of antigen in most tissues, and 5 - major accumulations of viral antigen in the leg tissues (Figure 2). Mosquitoes that received an IFA score of 5 were designated as super-infected (SI+), mosquitoes with IFA scores of 1-4 were designated as infected (I+), and mosquitoes with an IFA score of zero were designated as LACV negative (I-) (Figure 2).

### Virus isolation

SI+ and I+ mosquitoes, as identified by IFA, were triturated with a pellet pestle (Fisher Scientific, Pittsburgh, PA) in a 1.5 ml microcentrifuge tube containing 1 ml of minimal essential medium (MEM) (Invitrogen, Carlsbad, CA), 2% fetal bovine serum, 200 μg/ml penicillin/streptomycin, 200 μg/ml fungicide, 7.1 mM sodium bicarbonate, and 1× nonessential amino acids. The homogenate was centrifuged for 10 minutes at 500 × g to form a pellet.

Monolayers of Vero E6 cells were grown in six-well plates at 37°C in an atmosphere of 5% CO_2_. Supernatant from each centrifuged mosquito homogenate (200 μl) was added to one well of a six-well plate and incubated at 37°C for one hour. Following incubation, 5 ml of medium were added to each well. The isolation of LACV was revealed by the presence of cytopathic effects (CPE) five days post-infection [33].

### Titrations

The LACV titers of SI+ mosquitoes were determined by endpoint titration in Vero E6 cells [33]. The mosquito homogenates were serially diluted 10^-1 ^- 10^-6 ^and 200 μl of each dilution was added to one well of a 96 well plate. Five days post-infection, the endpoint was determined as the highest dilution causing CPE. The virus titer was calculated and expressed as log_10 _TCID_50_/ml [49].

### Plaque purification

Monolayers of Vero E6 cells in six-well plates were used for plaque purification of LACV isolates [33]. Virus isolates were serially diluted 10^-1 ^to 10^-6 ^and 200 μl of each virus dilution was added to one well and incubated at 37°C for 1 hour. The virus inoculum was then removed and 5 ml of overlay (1% agar in Medium 199, 10% fetal bovine serum, 7.1 mM sodium bicarbonate, 0.2% diethylaminoethyl-dextran in Hank's BSS, 1× Eagle basal medium vitamins, 1× Eagle basal amino acids) were added to the well. After six days of incubation at 37°C in 5% CO_2_, 200 μl of the detection solution, methylthiazolyldiphenyl-tetrazolium bromide (MTT, 5 mg/ml in PBS), were added to each well. The plates were incubated overnight and wells were then examined for plaques. Isolated plaques were individually picked and placed in 1 ml of MEM with 0.2% FBS for 1 h at 37°C. The eluted virus was added to a Vero cell monolayer in one well of a six-well plate. The cells were then incubated for five to seven days and the presence of virus was confirmed by CPE.

### RNA purification from virus isolates and amplification by reverse transcription PCR

Supernatant and cells from the wells containing plaque purified virus were removed and placed in a 15 ml conical tube and centrifuged at 3000 rpm for 10 minutes. The supernatant was removed, the cell pellet was resuspended in 500 μl of TRIzol, and total RNA was extracted according to manufacturer's instructions (Invitrogen, Carlsbad, CA). The entire S, M, and L RNA segments were transcribed to cDNA using Superscript II reverse transcriptase (Invitrogen, Carlsbad, CA) and amplified by PCR using Ex Taq DNA polymerase (Takara, Shiga, Japan) according to manufacturer's instructions. The S segment was amplified in two separate fragments, the M segment in three fragments, and the L segment in four fragments (Table 6). PCR was performed in a thermal cycler with the following program: 94°C for 5 min, 37 cycles of [94°C for 1 min., 57°C for 1 min. and 72°C for 2.5 min] followed by a final extension at 72°C for 8 min.

**Table 6:** Primer sets for amplification of the LACV RNA

Genome Segment	Primer	Primer Sequence (5'-3')	Primer position	Product Size (bp)
Primers used for the amplification of genome segments				

S-A	SF1	AGTAGTGTACCCCACTTGAATAC	1-23	525
	SR	CTTAAGGCCTTCTTCAGGTATTGAG	549-572	

S-B	SF3	CTTAAGGCCTTCTTCAGGTATTGAG	453-476	486
	SR1	AGTAGTGTGCCCCACTGAATAC	963-984	

M-C	MF1	AGTAGTGTACTACCAAGTATAGATGAACG	1-27	2211
	MR10	GACTCCTTTCCTCTAGCAAGG	2239-2258	

M-D	MF9	CAGACAACATGGAGAGTGTAC	1798-1818	1786
	MR5	GTCAAATCTGGGAACTCCATTGCC	3605-3628	

M-E	MF15	CAAGCTCATGGGGATGCGAAGAG	3249-3271	1235
	MR1	AGTAGTGTGCTACCAAGTATA	4507-4527	

L-F	LF1	AGTAGTGTACCCCTATCTACAAAAC	1-25	2955
	LR20	GTTTTCCCTCTGTTCGCACTC	2381-2401	

L-G	LF8A	CAACTTGCCTACTATTCAAAC	1899-1919	2164
	LR7	CCAATCCAACTGTACTAATCATTGAC	4084-4108	

L-H	LF8	GCTACCAGGGCAGTCAAATGACCC	3987-4011	1949
	LR10	CCTCTGCAACGTTAACTACACATACTG	5961-5986	

L-I	LF10	CAGATATTGTCTGGTGGCCATAAAGCC	5288-5314	1646
	LR12	AGTAGTGTGCCCCTATCTTC	6961-6980	

Primers used for the amplification of NSs and NSm				

S	NSsF-int	TTTGAAAATAAATTGTTGTTGACTG	24-48	472
	NSsR-int	CCCACTGTCCCATCCTACAC	476-495	

S	NSsF-ext	ACTCCACTTGAATACTTTGAAAATAAA	9-35	563
	NSsR-ext	CAATGGTCAGCGGGTAGAAT	552-571	

M	NSmF-int	CTGGATTGTGCCCTGGTTAT	918-937	598
	NSmR-int	TCAGTCTCTAGGCAGGTGGTG	1495-1515	

M	NSmF-ext	GCGGTGCTCGCTATGATACT	870-889	742
	NSmR-ext	AATTGGGTTGCAATGTTGGT	1592-1611	

### Sequencing

PCR products were separated by electrophoresis in 1% agarose gels with Tris-acetate-EDTA (TAE) buffer, stained with ethidium bromide, excised and extracted using the Powerprep Express Gel Extraction kit (Marligen Biosciences, Ijamsville, MD) according to manufacturer's instructions. PCR products were sequenced using the ABI PRISM dye terminator cycle sequencing kit (Applied BioSystems, Foster City, CA) and the ABI 310 DNA automated sequencer at Macromolecular Resources, CSU.

### Amplification of NSs and NSm genes of LACVs from SI+ and I+ mosquitoes

I+ and SI+ mosquitoes were triturated in 500 μl TRIzol LS reagent (Invitrogen, Carlsbad, CA), and total RNA was isolated from the homogenate per manufacturer's instructions. NSs and NSm genes were transcribed and PCR amplified in separate reactions using SuperScript™ III One-Step RT-PCR Kit with Platinum^® ^Taq (Invitrogen, Carlsbad, CA). External primers are listed in Table 6. The thermal cycling conditions for the RT-PCR protocol were: 55°C for 30 min, 94°C for 2 min, 40 cycles of [94°C for 30 s, 56°C for 30 s, and 68°C for 1 min] followed by a final extension at 68°C for 5 min. RT-PCR products were further amplified by nested PCR using Vent DNA polymerase (New England Biolabs, Ipswich, MA). Internal primers are listed in Table 6. The thermal cycling conditions were: 94°C for 2 min, 35 cycles of [94°C for 30 s, 56°C for 30 s, and 72°C for 75 s] followed by a final extension at 72°C for 5 min.

For virus isolates obtained from SI+ mosquitoes, 200 μl virus stock were added to 600 μl TRIzol LS reagent and viral RNA was isolated per manufacturer's instructions. NSs and NSm were transcribed and PCR amplified in separate reactions. Primers used in these reactions were the same as those used for the nested PCR step of I+ and SI+ samples. A nested PCR step was unnecessary and not applied to SI+ isolates. PCR amplicons were purified with QIAquick PCR Purification Kit (Qiagen, Valencia, CA) and sequenced bi-directionally as above. Sequences were assembled and aligned using ContigExpress and AlignX software (Invitrogen, Carlsbad, CA).

### Phylogenetic analyses

LACV was isolated from five SI+ mosquitoes and RNA was extracted from the isolates, amplified by RT-PCR, and sequenced. The entire S, M and L segments were sequenced and analyzed. Previously published LACV sequences [50-54] were obtained from GenBank. The viruses used in this analysis and their respective GenBank Accession numbers are listed in Table 3.

Maximum Likelihood (ML) trees [55] were derived for all three segments and for the NSm dataset using RAxML (Randomized Axelerated Maximum Likelihood) [56,57]. RAxML performs sequential and parallel ML based inference of large phylogenetic trees. RAxML was initially used to generate five fixed starting Maximum Parsimony (MP) trees and these were then used as starting trees to estimate ML trees using a fixed setting with GTRMIX and then using the automatically determined setting on the same five starting MP trees. We evaluated the final trees under GTRGAMMA to compare the final likelihoods. The conditions that yielded the best likelihood scores were used in further analyses. Next the optimal number of parameters in the ML models was determined by increasing the number of parameters by increments of 5 up to 55. Again GTRGAMMA was used to compare the final likelihoods. GTRMIX located the Best-Known Likelihood (BKL) tree and GTRCAT performed bootstrapping with 100 pseudo-replications. This generated a bipartitions Newick treefile with information on ML topology, rates and bootstrap support. This bipartitions Newick file was loaded into TreeGraph2 http://treegraph.bioinfweb.info/ to produce the final phylogeny graphics.

### Analyses of molecular evolution

DNAsp5.0 [58] was used to assign coding and noncoding regions in the S, M and L segments and to subdivide each sequence dataset into previously published versus SI+ groups. DNAsp5.0 then computed in each group the number of segregating sites (S), unique haplotypes (Hap), and singletons η_e_. Nucleotide diversity (π - Nei [36]), π_s _among synonymous sites, π_a _among replacement sites, θ/comp [36], and F* [59] were also computed with DNAsp5.0.

F* is a normalized comparison of shared substitutions (η_i_) relative to those appearing once, "singletons" (η_e_). While no explicit examination of a genealogy is involved, the F* test assumes that singletons arose recently and appear on the tips of branches while shared mutations (η_i_) are older and arose internally on nodes of the genealogy. When alleles are maintained at frequencies that approximate the average mutation rate then F* = 0 and polymorphisms are assumed to evolve in a manner predicted by neutral evolution. Fu and Li [59] pointed out that F* = 0 does not imply no selection because the neutral model implicitly assumes that the majority of novel alleles are deleterious and removed through purifying selection. In contrast, F* < 0 values reflect an excess of singletons (η_e_) in a population and this pattern is consistent with positive selection wherein genetic variants sweep throughout a population. Conversely, F* >0 values indicate an excess of intermediate-frequency η_i _alleles and can result from balancing selection or disruptive selection maintaining alleles at frequencies greater than the average mutation rate. DNAsp5.0 was also used to compute confidence intervals of F* by simulations using the coalescent algorithm and to compute differences in nucleotide frequency (D_a _- Nei [36]) between previously published and SI+ isolates across the LACV genome and gene sequences using the Sliding Window method.

Relative Synonymous Codon Usage (RSCU) was analyzed within and among previously published and SI+ isolates using the GCUA (General Codon Usage Analysis) package [35]. The program was subsequently used to perform three analyses on the codon usage tables: 1) correspondence analysis (CA) [60]), 2) Principal Components Analysis (PCA) and 3) a cluster analysis based upon McInerney's distance measure [35], based on the differences in RSCU values.

## Competing interests

The authors declare that they have no competing interests.

## Authors' contributions

SMR conducted mosquito rearing from field collected liners, mosquito IFA, PCR, and virus isolation assays, and phylogenetic analyses. ECM conducted LACV NSs and NSm amplifications from SI+ and I+ mosquitoes and analyzed NT and AA variability in the respective phenotypes. MKB participated in mosquito rearing and IFA phenotyping of mosquitoes and in manuscript preparation and submission. ETB first detected SI+ mosquitoes and participated in early studies to characterize the phenotypes. DG supervised all field crews for identifying field sites, collecting of *Ae. triseriatus *eggs and processing and shipping of oviposition liners to Colorado. CDB and BJB were co-PIs of the NIH grant that made these studies possible, established the overall studies and experimental design, supervised laboratory and virologic investigations, analyzed data, and prepared the manuscript. WCB over saw and conducted molecular evolution and phylogenetic analyses and prepared the manuscript. All authors participated in the preparation of this manuscript and approve its submission to Virology Journal.

## Supplementary Material

Additional file 1 **Figure **1. Nucleotide sequence of LACV NSs from SI+ and I+ mosquitoes from one site. The NT sequence is absolutely conserved in all NSs genes analyzed. Click here for file
