# Supplementary material for: Identification of super-infected Aedes triseriatus mosquitoes collected as eggs from the field and partial characterization of the infecting La Crosse viruses
Source: Virol J. 2010 Apr 22;7:76. doi: 10.1186/1743-422X-7-76 (PMC2873378; doi:10.1186/1743-422X-7-76)
Supplement: Additional file 1 — Figure 1. Nucleotide sequence of LACV NSs from SI+ and I+ mosquitoes from one site. The NT sequence is absolutely conserved in all NSs genes analyzed. [file 1743-422X-7-76-S1.DOC]

**Additional Files – Figure 1.**

1 100

I+LAC05/KBT/Monroe,WI/m/07A (1) ATGATGTCGCATCAACAGGTGCAAATGGATTTGATCCTGATGCAGGGTATATGGACTTCTGTGTTAAAAATGCAGAATCACTCAACCTTGCTGCAGTTAG

I+LAC06/GOLF/Houston,MN/m/07A (1) ....................................................................................................

I+LAC07/HIDV/Winona,MN/m/07A (1) ....................................................................................................

I+LAC08/NAT/Crawford,WI/m/07B (1) ....................................................................................................

I+LAC09/HIDV/Winona,MN/m/07B (1) ....................................................................................................

I+LAC10/NAT/Crawford,WI/m/07C (1) ....................................................................................................

I+LAC11/NAT/Crawford,WI/m/07D (1) ....................................................................................................

I+LAC12/LCVP/Houston,MN/m/07A (1) ....................................................................................................

I+LAC13/ALP/LaCrosse,WI/m/07A (1) ....................................................................................................

I+LAC14/DAKE/Winona,MN/m/07A (1) ....................................................................................................

SI+LAC01/SVP/LaCrosse,WI/m/06A (1) ....................................................................................................

SI+LAC03/NAT/Crawford,WI/m/07A (1) ....................................................................................................

SI+LAC16/NAT/Crawford,WI/m/07B (1) .................................................................................................... SI+LAC19/BEN2/Lafayette,WI/m/07D (1) ....................................................................................................

SI+LAC20/BEN2/Lafayette,WI/m/07E (1) ....................................................................................................

SI+LAC21/BEN2/Lafayette,WI/m/07F (1) ....................................................................................................

SI+LAC22/CAL-GA/Houston,MN/m/07G (1) ....................................................................................................

SI+LAC23/CAL-GA/Houston,MN/m/07J (1) ....................................................................................................

SI+LAC24/CAL-GA/Houston,MN/m/07K (1) ....................................................................................................

Consensus (1) ATGATGTCGCATCAACAGGTGCAAATGGATTTGATCCTGATGCAGGGTATATGGACTTCTGTGTTAAAAATGCAGAATCACTCAACCTTGCTGCAGTTAG

101 200

I+LAC05/KBT/Monroe,WI/m/07A (101) GATCTTCTTCCTCAATGCCGCAAAGGCCAAGGCTGCTCTCTCGCGTAAGCCAGAGAGGAAGGCTAACCCTAAATTTGGAGAGTGGCAGGTGGAGGTTATC

I+LAC06/GOLF/Houston,MN/m/07A (101) ....................................................................................................

I+LAC07/HIDV/Winona,MN/m/07A (101) ....................................................................................................

I+LAC08/NAT/Crawford,WI/m/07B (101) ....................................................................................................

I+LAC09/HIDV/Winona,MN/m/07B (101) ....................................................................................................

I+LAC10/NAT/Crawford,WI/m/07C (101) ....................................................................................................

I+LAC11/NAT/Crawford,WI/m/07D (101) ....................................................................................................

I+LAC12/LCVP/Houston,MN/m/07A (101) ....................................................................................................

I+LAC13/ALP/LaCrosse,WI/m/07A (101) ....................................................................................................

I+LAC14/DAKE/Winona,MN/m/07A (101) ....................................................................................................

SI+LAC01/SVP/LaCrosse,WI/m/06A (101) ....................................................................................................

SI+LAC03/NAT/Crawford,WI/m/07A (101) ....................................................................................................

SI+LAC16/NAT/Crawford,WI/m/07B (101) ....................................................................................................

SI+LAC19/BEN2/Lafayette,WI/m/07D (101) ....................................................................................................

SI+LAC20/BEN2/Lafayette,WI/m/07E (101) ....................................................................................................

SI+LAC21/BEN2/Lafayette,WI/m/07F (101) ....................................................................................................

SI+LAC22/CAL-GA/Houston,MN/m/07G (101) ....................................................................................................

SI+LAC23/CAL-GA/Houston,MN/m/07J (101) ....................................................................................................

SI+LAC24/CAL-GA/Houston,MN/m/07K (101) ....................................................................................................

Consensus (101) GATCTTCTTCCTCAATGCCGCAAAGGCCAAGGCTGCTCTCTCGCGTAAGCCAGAGAGGAAGGCTAACCCTAAATTTGGAGAGTGGCAGGTGGAGGTTATC

201 279

I+LAC05/KBT/Monroe,WI/m/07A (201) AATAATCATTTTCCTGGAAACAGGAACAACCCAATTGGTAACAACGATCTTACCATCCACAGATTATCTGGGTATTTAG

I+LAC06/GOLF/Houston,MN/m/07A (201) ...............................................................................

I+LAC07/HIDV/Winona,MN/m/07A (201) ...............................................................................

I+LAC08/NAT/Crawford,WI/m/07B (201) ...............................................................................

I+LAC09/HIDV/Winona,MN/m/07B (201) ...............................................................................

I+LAC10/NAT/Crawford,WI/m/07C (201) ...............................................................................

I+LAC11/NAT/Crawford,WI/m/07D (201) ...............................................................................

I+LAC12/LCVP/Houston,MN/m/07A (201) ...............................................................................

I+LAC13/ALP/LaCrosse,WI/m/07A (201) ...............................................................................

I+LAC14/DAKE/Winona,MN/m/07A (201) ...............................................................................

SI+LAC01/SVP/LaCrosse,WI/m/06A (201) ...............................................................................

SI+LAC03/NAT/Crawford,WI/m/07A (201) ...............................................................................

SI+LAC16/NAT/Crawford,WI/m/07B (201) ............................................................................... SI+LAC19/BEN2/Lafayette,WI/m/07D (201) ............................................................................... SI+LAC20/BEN2/Lafayette,WI/m/07E (201) ............................................................................... SI+LAC21/BEN2/Lafayette,WI/m/07F (201) ...............................................................................

SI+LAC22/CAL-GA/Houston,MN/m/07G (201) ...............................................................................

SI+LAC23/CAL-GA/Houston,MN/m/07J (201) ...............................................................................

SI+LAC24/CAL-GA/Houston,MN/m/07K (201) ...............................................................................

Consensus (201) AATAATCATTTTCCTGGAAACAGGAACAACCCAATTGGTAACAACGATCTTACCATCCACAGATTATCTGGGTATTTAG
